# Using machine learning to find genes associated with sudden death

**DOI:** 10.3389/fcvm.2022.1042842

**Published:** 2022-10-25

**Authors:** Kena Zhou, Congbo Cai, Yi He, Zhihua Chen

**Affiliations:** ^1^Department of Gastroenterology, Ningbo No. 9 Hospital, Ningbo, China; ^2^Department of Emergency, Yinzhou No. 2 Hospital, Ningbo, China; ^3^Department of Emergency, Ningbo First Hospital, Ningbo, China

**Keywords:** sudden death, machine learning, molecular autopsy, characteristic genes, biomarkers

## Abstract

**Objective:**

To search for significant biomarkers associated with sudden death (SD).

**Methods:**

Differential genes were screened by comparing the whole blood samples from 15 cases of accidental death (AD) and 88 cases of SD. The protein-protein interaction (PPI) network selects core genes that interact most frequently. Machine learning is applied to find characteristic genes related to SD. The CIBERSORT method was used to explore the immune-microenvironment changes.

**Results:**

A total of 10 core genes (MYL1, TNNC2, TNNT3, TCAP, TNNC1, TPM2, MYL2, TNNI1, ACTA1, CKM) were obtained and they were mainly related to myocarditis, hypertrophic myocarditis and dilated cardiomyopathy (DCM). Characteristic genes of MYL2 and TNNT3 associated with SD were established by machine learning. There was no significant change in the immune-microenvironment before and after SD.

**Conclusion:**

Detecting characteristic genes is helpful to identify patients at high risk of SD and speculate the cause of death.

## Introduction

Sudden death (SD) is the sudden, non-violent death of a healthy or seemingly healthy person caused by an outbreak of disease or an underlying disease in the body. Those who died within 24 h after the onset of symptoms is called SD. It is common in young or middle-aged adults, which imposes a significant burden on families and society.

The diagnosis of the cause of SD is usually based on autopsy (1). Even with the development of forensic science, there is still a considerable reasons of SD that cannot be inferred (2). With the deepening of research, it is found that genetic factors play a crucial role in SD (3). It is estimated that up 35% of sudden unexplained death cases are associated with genetic variants in cardiac channels (4). With the development of gene sequencing technology, molecular autopsy is gradually used for forensic identification. This method is especially suitable for SD of unknown causes (5, 6). However, molecular autopsy is still in its infancy, with only preliminary testing in patients with a genetic family history (7). There is still a lack of research on specific genes related to SD.

The Genotype-Tissue Expression (GTEx) database holds data of normal tissue DNA and RNA sequencing (RNA-seq) from donors (8). Now 54 tissues from 948 donors have been preserved, including 17,382 samples. Donor death time in the database were divided into instantaneous death (0 h), short-term death (0–1 h), moderate death (1–24 h) and slow death (> 24 h). This provides a good source of data source for the study of the causes of SD. It can assist forensic medicine to find characteristic genes related to SD.

Machine learning is a collection of data-analytical techniques aimed at building predictive models from multi-dimensional datasets (9). Machine learning outperforms traditional statistical algorithms when faced with complicated problems involving a large number of noisy and heterogeneous predictor (10). It is becoming an integral part of modern data mining and clinical diagnosis (11).

In this study, we searched for the characteristic genes of SD by machine learning based on the GTEx database. These biomarkers can be used to screen patients at high risk of SD. And also characteristic genes provide potential advice for taking early measures in high-risk patients. In addition, theoretical support for molecular autopsy can also be verified.

## Materials and methods

### Datasets

Donor RNA-seq was downloaded from the GTEx (RRID:SCR_013042) and all sequencing results were normalized by FPKM. The relevant clinical information of the donors can be downloaded from the GTEx official website.^1^ The GTEx emphasizes that the database is free and open to the society, but the official website information needs to be marked in the paper. Database use does not require institutional review board approval and informed consent.

### Differential gene screening and protein-protein interaction network analysis

The Wilcox test in the “limma” package was used to screen significantly differentially expressed genes between AD and SD in GTEx cohort. We took | LogFC| > 1, false discovery rate (FDR) < 0.05 as the threshold point for differential genes. Simultaneously, volcano plots and heatmaps of differential genes were figured out. There is a close relationship between the biological functions of gene/protein clusters (12). Therefore, proteins usually cooperate to perform biological functions. The protein-protein interaction (PPI) network helps to differentiate the core genes in SD according to the frequency of interaction. PPI analysis was performed on the STRING database^2^ with a confidence index of 0.7. The more the interaction relationship, the more important role the gene plays in the process of SD. The connectivity table was drawn in R language, and connectivity ≥ 5 is defined as core genes.

### Biological role and disease analysis

Function, pathway enrichment and disease analysis of core genes based on “clusterProfiler,” “enrichplot,” “org.Hs.eg.db,” “ggplot2,” “GSEABase” and “DOSE” packages were performed in R language. The biological significance of core genes was analyzed by Gene Ontology (GO) functional enrichment, including Biological Process (BP), Cellular Components (CC), and Molecular Function (MF). Kyoto Encyclopedia of Genes and Genomes (KEGG) enrichment analysis was used to explore the pathways of core genes. Disease Ontology (DO) enrichment analysis was applied to discover major diseases led by core genes. *P* < 0.05 and corrected *P* < 0.05 were considered to be statistically significant in all the analysis process. The visualization of GO, KEGG, and DO could be achieved by the R package “GOplot.”

### Machine learning

In order to reduce errors, we used two different machine learning algorithms to seek for potential characteristic genes. The Least Absolute Shrinkage and Selection operator (LASSO) is a machine learning based regression analysis algorithm that uses regularization to remove highly correlated genes, which can avoid overfitting.

Support vector machine recursive feature elimination (SVM-RFE) is a machine learning algorithm based on classification and regression. Gene redundancy can be automatically eliminated and a better, more compact subset of genes can be generated. We use the R packages of “glmnet” and “e1071” to implement machine learning algorithms for LASSO and SVM-RFE. Finally, characteristic genes are obtained by intersection.

### Analysis of clinical value of characteristic genes

To test the diagnostic value of the characteristic genes, we compared the expression of characteristic genes in AD and SD groups in R language software. Moreover, receiver operating characteristic (ROC) curves were drawn to analyze the validity of the characteristic genes.

### Analysis of the expression of characteristic genes in human tissues

Human anatomy were drawn in R software based on “gganatogram,” “dplyr,” “viridis,” and “gridExtra.” The R package of “gganatogram” can draw modular anatomical maps and quantify the expression of characteristic genes in various tissues in human body. The Human Protein Atlas (HPA) database^3^ was used to validate the protein expression level of the target SD genes.

### Analysis of immune-microenvironment

We used the CIBERSORT (RRID:SCR_016955) algorithm to assess the relative proportions of immune cell infiltration in different populations. And the abundance of 22 immune cells can be quantified *via* this method. The R package of “corrplot” visualizes 22 types of immune cells. And the R package of “vioplot” draws violin plots to show differences in immune cell infiltration between different groups.

## Results

### Clinical information

The clinical information of the patients was obtained on the GTEx official website (see text footnote 1). The AD group consisted of 15 whole blood samples from 15 donors who died unexpectedly (violent and fast death). The SD group included 88 blood samples, including 69 donors with fast death of natural causes (0–1 h) and 17 donors with intermediate death (1–24 h). More details could be referred in Table 1.

**TABLE 1:** Summary of clinical information for donors.

Characteristic	Accidental death (*N* = 15)	Sudden death (*N* = 86)
**Age**		
<30	5	3
≥30	10	83
**Gender**		
Male	11	66
Female	4	20
**Death circumstances**		
D1	15	0
D2	0	69
D3	0	17
D4	0	0

Death circumstances: D1, Violent and fast death; D2, Fast death of natural causes; D3, Intermediate death; D4, Slow death.

### Analysis of protein-protein interaction network for differential genes

**FIGURE 1:**
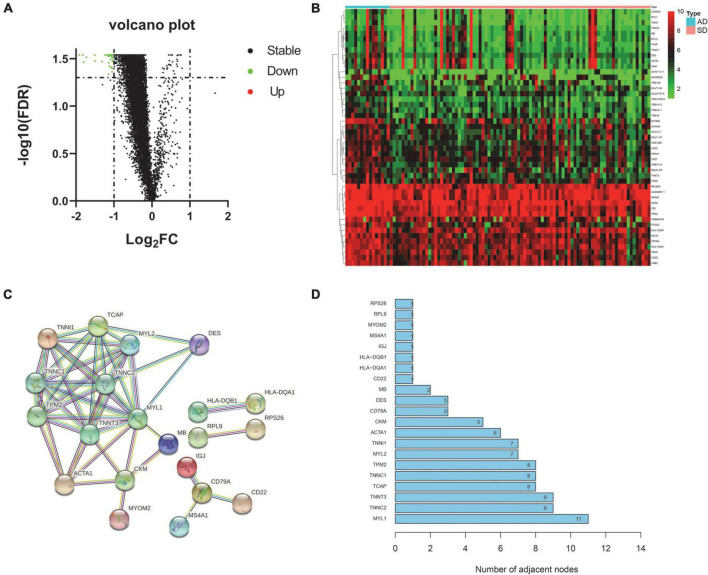
Differential expression and core gene screening. **(A)** Volcano plot of all genes. (Red dots represent up-regulated genes and green dots represent down-regulated genes). **(B)** Heatmap of differential genes in AD and SD groups. (Rows represent 47 differential genes and columns represent samples). **(C)** PPI network of differential genes. (Nodes represent hub genes. Lines represent interactions between hub genes). **(D)** Bar graph of all hub genes in the PPI network. (The x-axis represents channel counts. The y-axis represents hub genes). AD, Accidental death; SD, Sudden death.

This study retrospectively analyzed whole blood samples from donors of AD and SD. According to the cutoff value, a total of 47 differential genes were obtained and considered to be related to SD (Supplementary Table 1). All of these genes were down-regulated in the SD group (Figure 1A). The heat map shows the expression levels of all differential genes in different groups (Figure 1B). To better understand the interactions between these SD-related genes, we used the STRING online database^4^ to construct a PPI network for 47 differential genes (Figure 1C). Ten genes with high interaction were identified as core genes (MYL1, TNNC2, TNNT3, TCAP, TNNC1, TPM2, MYL2, TNNI1, ACTA1, CKM). It was suggested that they play an important role in SD process (Figure 1D).

### Functional correlation analysis of core genes

**FIGURE 2:**
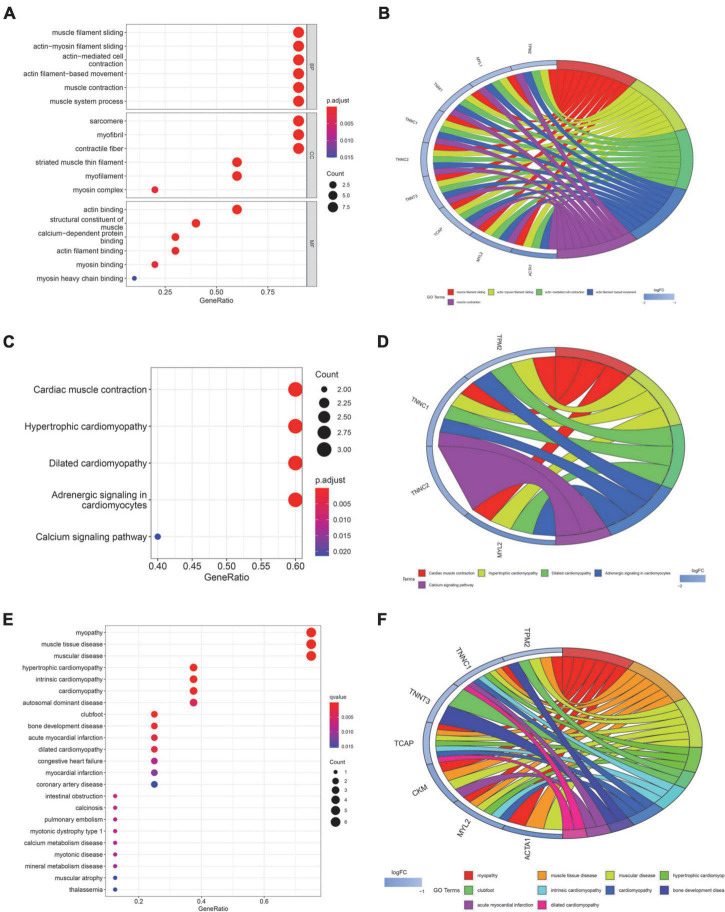
Function, pathway, and disease enrichment analysis of core genes. **(A)** Bubble plot of GO function enrichment for core genes. (BP, Biological Process; CC, Cellular Components; MF, Molecular Function). **(B)** Circle plot of GO functional enrichment. **(C)** Bubble map of KEGG pathway enrichment for core genes. **(D)** Circle plot of KEGG enrichment analysis. **(E)** Bubble plot of DO enrichment for core genes. **(F)** Circle plot of DO enrichment analysis. The size of bubbles in the bubble plot represents the number of core genes in the corresponding pathway. The color of the bubbles represents the adjusted *p*-value. The circle plot illustrates certain core genes corresponding to the GO/KEGG terminology or disease. LogFC represents the expression level of gene.

In order to explore the role of these genes in the process of SD and related diseases. We focused on the function, pathway and disease analysis of 10 core genes related to SD. GO analysis results shows that the annotations of genes come from three ontologies, namely biological process (BP), molecular function (MF), and cellular component (CC). BP terminology mainly contains muscle filament sliding, muscle contraction. MF terminology mainly contains sarcomere, myofibril, contractile fiber. CC terminology mainly contains actin binding, myosin binding (Figure 2A). The circle diagram shows that core genes are mainly enriched in muscle contraction, actin filament-based movement, muscle filament sliding, etc. (Figure 2B). The pathways of core genes were mainly enriched in Cardiac muscle contraction, Hypertrophic cardiomyopathy (HCM), Dilated cardiomyopathy (DCM), Adrenergic signaling in cardiomyocytes, and Calcium signaling pathway (Figure 2C). The circle diagram shows certain core genes corresponding to KEGG pathways (Figure 2D). DO analysis shows that the core genes of SD were mainly enriched in myopathy, HCM, cardiomyopathy, autosomal dominant disease, clubfoot, acute myocardial infarction, DCM, pulmonary embolism, and other diseases (Figure 2E). The circle diagram shows top 10 diseases corresponding to SD-associated core genes (Figure 2F).

### Machine learning characteristic genes

**FIGURE 3:**
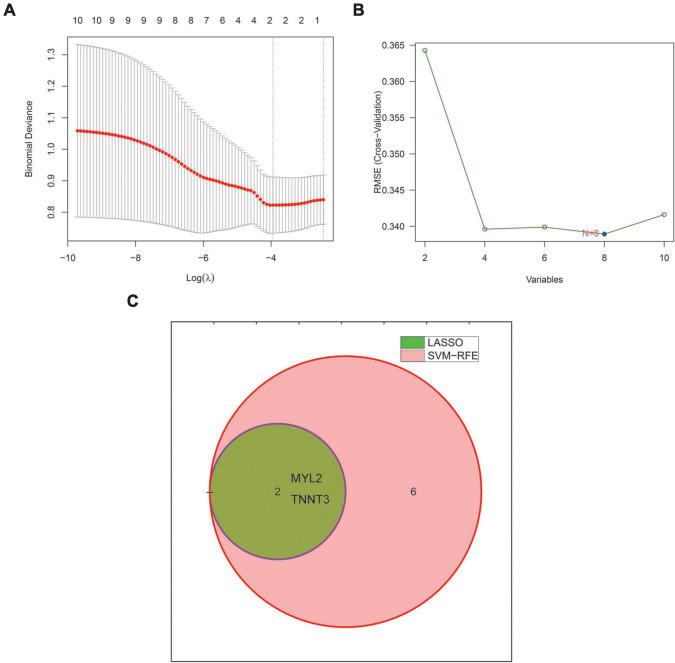
Machine learning identifies characteristic genes of sudden death. **(A)** The LASSO regression algorithm was used to select the characteristic genes of sudden death. **(B)** SVM-RFE algorithm to select the characteristic genes of sudden death. (The blue point represents the lowest error rate, correspondingly to the best genome selected by SVM-RFE). **(C)** Venn diagram showing 2 sudden death characteristic genes shared by LASSO (green) and SVM-RFE (pink) algorithms. LASSO, least absolute shrinkage and selector operation. SVM-RFE, support vector machine-recursive feature elimination.

We used two machine learning methods, LASSO regression and SVM-RFE, to study the core genes of SD. LASSO regression learned from the 10 core genes to obtain 2 characteristic genes of SD (Figure 3A). The SVM-RFE algorithm learned from 10 core genes to obtain 8 characteristic genes of SD (Figure 3B). The two algorithms were intersected by a Venn diagram, and 2 common genes were obtained as the characteristic genes closely related to SD (Figure 3C).

### Analysis of clinical value of characteristic genes

**FIGURE 4:**
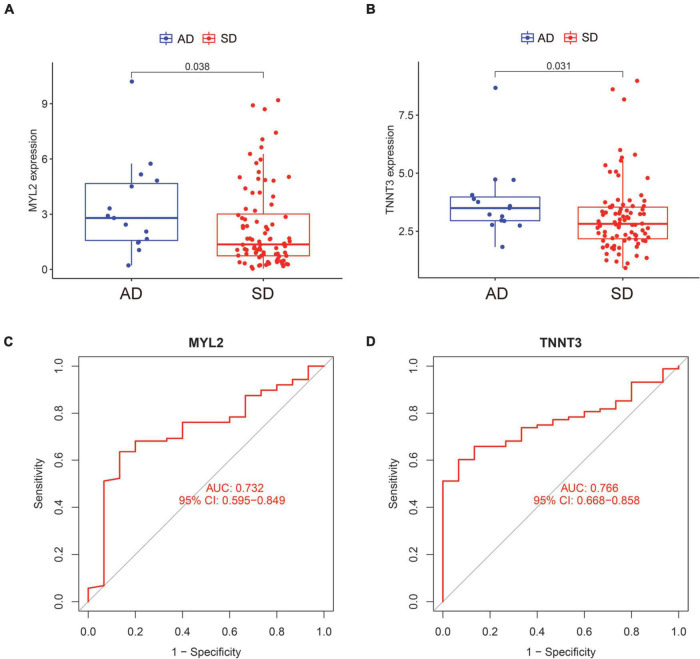
Expression and ROC curves of characteristic genes of sudden death. **(A)** The expression level of MYL2 in AD and SD groups. **(B)** The expression level of TNNT3 in AD and SD groups. **(C)** The ROC curve of MYL2. **(D)** The ROC curve of TNNT3. ROC, receiver operating characteristic; AD, Accidental death; SD, Sudden death.

We compared the expression of the two characteristic genes in the AD and SD groups. And the ROC curve was exhibited to confirm the clinical value of the characteristic genes. The expression of SD-related characteristic genes (MYL2 and TNNT3) were both decreased in the SD group (Figures 4A,B; all *P* < 0.05). The AUC value of ROC curves for MYL2 was 0.732 (95%CI = 0.595–0.849) (Figure 4C). The AUC value for TNNT3 was 0.766 (95%CI = 0.668–0.858) (Figure 4D). These shows that the SD-associated characteristic genes have good performance with high diagnostic ability.

### Expression analysis of characteristic genes in human body

**FIGURE 5:**
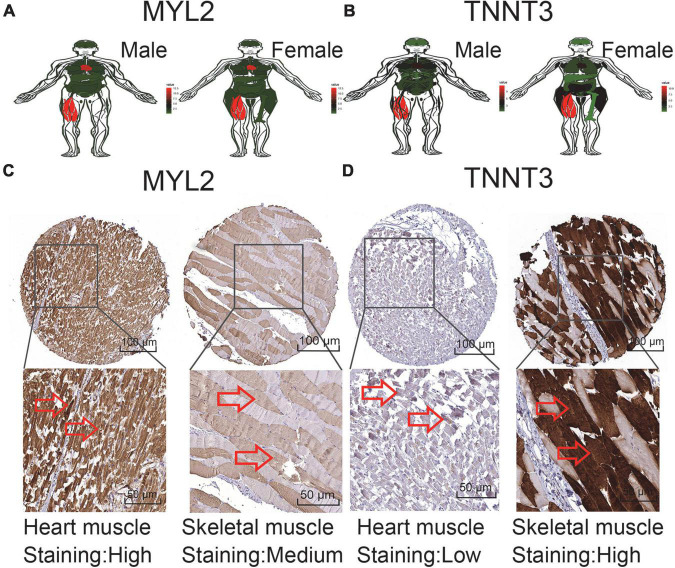
Expression of sudden death characteristic genes in human tissues. **(A,B)** MYL2 and TNNT3 expression levels in tissues in males and females. **(C)** Validation of MYL2 in turquoise module by HPA (IHC). **(D)** Validation ofTNNT3 in turquoise module by HPA (IHC). Red represents high expression, green represents low expression, and black represents mediate expression.

In order to verify the expression of characteristic genes in the human body, we extracted the expression levels of MYL2 and TNNT3 in various tissues from GTEx database. And an anatomical map was generated (Figures 5A,B). Moreover, the protein levels of immunohistochemistry (IHC) staining obtained from the HPA database illustrated that MYL2 was highly expressed in cardiac muscle and moderately expressed in skeletal muscle (Figure 5C); while TNNT3 is lowly expressed in cardiac muscle and highly expressed in skeletal muscle (Figure 5D).

### Immune infiltration analysis

**FIGURE 6:**
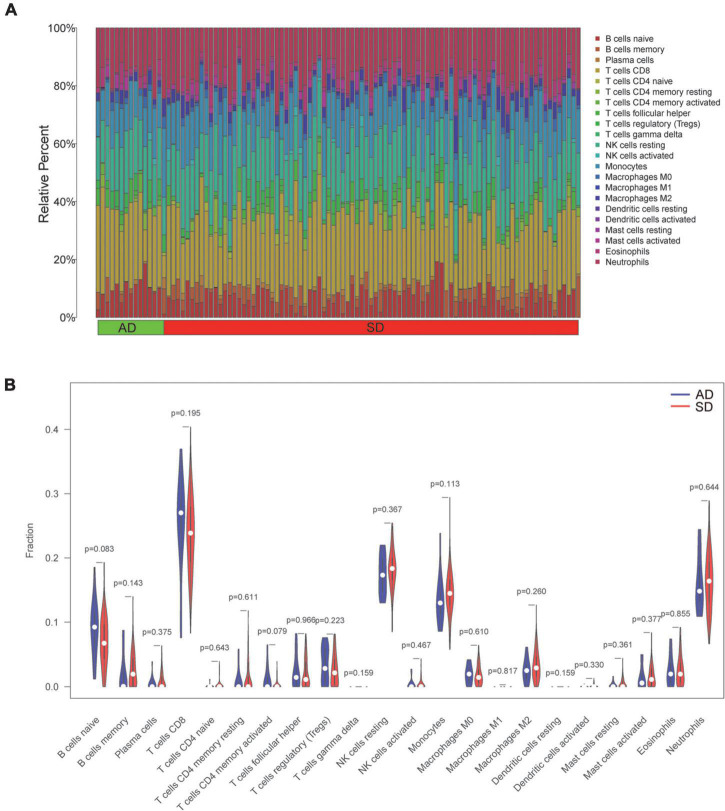
Profile and visualization of immune cell infiltration. **(A)** The infiltration of 22 immune cells after quantification by the CIBERSORT algorithm. (The X-axis represents the sample and the Y-axis shows the percentage of 22 immune cells in the sample as stacked bars). **(B)** Violin plot showing comparison based on 22 immune cells. (Blue and red represent AD and SD group samples, respectively). AD, Accidental death; SD, Sudden death.

We explored immune cell profiles in patients in AD and SD groups using the CIBERSORT method. The infiltration of 22 immune cells were estimated in SD and AD groups in Figure 6A. The ratios of 22 immune cells were further compared in SD and AD groups (Figure 6B). The results showed that all immune cell differences were not statistically significant (*P* > 0.05). This suggested that although SD was caused by various diseases, no significant participation of immune cells was witnessed in this short-term process.

## Discussion

SD is the most serious clinical adverse phenomenon. Accurate cause of death is difficult to conclude even with the aid of forensic science. Based on the large-scale database of the GTEx platform, we explore the related genes and diseases that cause SD. We screened out 10 core genes (MYL1, TNNC2, TNNT3, TCAP, TNNC1, TPM2, MYL2, TNNI1, ACTA1, CKM) from the database. Two characteristic genes (MYL2, TNNT3) were extruded *via* two machine learning algorithms, with good diagnostic ability. Our study demonstrated that most sudden deaths are acute onsets of chronic diseases without the involvement of the immune microenvironment.

We are the first to put forward 10 core genes related to SD *via* the GTEx database. The biological processes of these core genes mainly focus on myofilament and sarcomere activities mediated by actin and myosin. Consistent with our study, Klaassen et al. pointed out that sarcomeric protein gene defects can cause various heart diseases (13). Furthermore, the possible causes of SD proposed from the core genes are as follows: myopathy, HCM, cardiomyopathy, autosomal dominant disease, clubfoot, acute myocardial infarction, DCM, pulmonary embolism, etc. These above diseases are clinically common and can lead to death in a short time. The major forms of cardiomyopathy include hypertrophic, dilated, restrictive and arrhythmogenic cardiomyopathy (14). Among them, HCM is usually witnessed with obvious heredity (15). At present, more and more scholars have pointed out the importance of gene detection in HCM risk stratification (16). Our study identified TNNC1, TCAP and MYL2 as the risk genes for SD in HCM. The mutation rate of TNNC1 in HCM patients is approximately 0.4% (17). Multiple studies have shown that mutations in TNNC1 cause HCM and early sudden cardiac death (18, 19). TCAP is a key regulator of muscle growth, and reduced TCAP expression will destroy muscle growth (20). MYL2 is also a risk gene for HCM, and Arg58Gln and R58Q mutations in MYL2 can lead to early sudden cardiac death (21, 22). This is consistent with our research. DCM is a type of cardiomyopathy characterized by left ventricular enlargement and systolic dysfunction. Our study showed that the main SD-related genes in DCM were TCAP and TNNC1. TCAP mutation was detected in DCM patients (23, 24). But whether the mutation of this gene can cause SD in DCM patients has not been reported in the literature. TNNC1 is also a risk gene for DCM (25). Numerous articles have reported premature sudden cardiac death or heart transplantation would occur in DCM patients with TNNC1-mutated (26, 27). Abnormal expression of core genes in various diseases will lead to the increase of SD rate. Focusing on core genes in hereditary diseases is helpful for the early identification and prevention of deadly outcomes.

Machine learning can discover excellent prognostic genes in the form of self-learning. MYL2 and TNNT3 were extraordinary extruded after machine learning. MYL2 is mainly expressed in the ventricle, and its mutation will cause HCM (28, 29). Statistics found that the probability of MYL2 mutation in HCM patients was 2.1–5% (28, 30–32). Manivannan et al. suggested that mutation in MYL2 in HCM families had resulted in SD of four children before the age of one (29). When MYL2 mutation existed in HCM patients, the clinical lesions appear early, the disease is severe, the prognosis is very poor, and many suffer early SD (21, 33). This is supportive to our study. Thus we advocate that genetic disease guidance can focus on SD associated genes.

Mutations in TNNT3 will cause various muscle disorders, mainly covering distal arthrogryposis (DA) (34). Also there are nemaline myopathy (NEM) (35) and atrial septal defect (36) associated with TNNT3 mutation. DA is a clinically and genetically heterogeneous disease, mainly characterized by congenital spasticity of the joints of the extremities. In 2018, Sandaradura et al. described that TNNT3 mutations had led to non-invasive ventilation in the neonatal period with a result of death at 8 months of age (37). Our study showed that TNNT3 was highly expressed in the heart, as well as in muscle tissues. Therefore, we consider that TNNT3 mutation would cause changes in the myocardium resulting SD. Although these two characteristic genes have less variation in other diseases, the probability of SD is greatly increased with their mutation. Early or aggressive clinical interventions such as heart transplantation or ICD are strongly suggested with characteristic genetic variants.

The AUC values of MYL2 and TNNT3 were 0.732 and 0.766, respectively. We consider that the final result of SD is caused by a large category of SD-related diseases. A single gene can only represent one or several diseases, not all diseases, so the AUC value is not very high. Our study also showed that no changes in the immune-microenvironment before and after death in SD patients. We supposed that SD is the result of a short-term deterioration of the disease without the involvement of immune cells.

This study has some limitations. First, most SD donors died in less than an hour. The main cause of death in these patients is sudden cardiac death, so the characteristic genes are relatively close to the genes related to cardiac death. Second, in order to protect the privacy of donors, GTEx platform only provides the age and gender of the donors, and no other specific clinical data was displayed. Therefore, valid information such as previous diseases, family history and autopsy cannot be obtained in details. Third, there might be bias in our study due to limited sample size, even if we used the PPI network to capture the most active genes as many as possible. Hopefully, we are looking forward to larger cohorts in future validation researches that may require multi-institutional collaboration.

## Conclusion

SD is caused by a variety of diseases, most of which are heart disease. Studies have shown that genetics play an important role in SD. Our study found that the cause of SD might be HCM, dilated cardiomyopathy, acute myocardial infarction, pulmonary embolism and so on. MYL2 and TNNT3 were discovered as characteristic genes by machine learning, which could predict the prognosis of SD. For high risk patients with familial SD history, the expression of SD genes can be investigated. For high risk patients, early intervention can be carried out, such as early cardiac surgery or pacemaker placement.

## Data availability statement

The datasets presented in this study can be found in online repositories. The names of the repository/repositories and accession number(s) can be found below: www.gtexportal.org.

## Ethics statement

Ethical review and approval was not required for the study on human participants in accordance with the local legislation and institutional requirements. Written informed consent for participation was not required for this study in accordance with the national legislation and the institutional requirements.

## Author contributions

KZ and ZC contributed to the study conception and design. CC and YH performed the material preparation, data collection, and analysis. KZ and CC wrote the first draft of the manuscript. ZC supervised the whole study. All authors read and approved the final manuscript.

## Footnotes

1
www.gtexportal.org/

2
https://www.string-db.org/

3
https://www.proteinatlas.org/

4
http//:string-db.org

## Supplementary material

Supplementary Figure 1 Graphical abstract.

Supplementary Table 1 47 differentiated genes associated with SD.
