# Supplementary figures and images for: Using machine learning to find genes associated with sudden death
Source: Front Cardiovasc Med. 2022 Oct 25;9:1042842. doi: 10.3389/fcvm.2022.1042842 (PMC9641215; doi:10.3389/fcvm.2022.1042842)

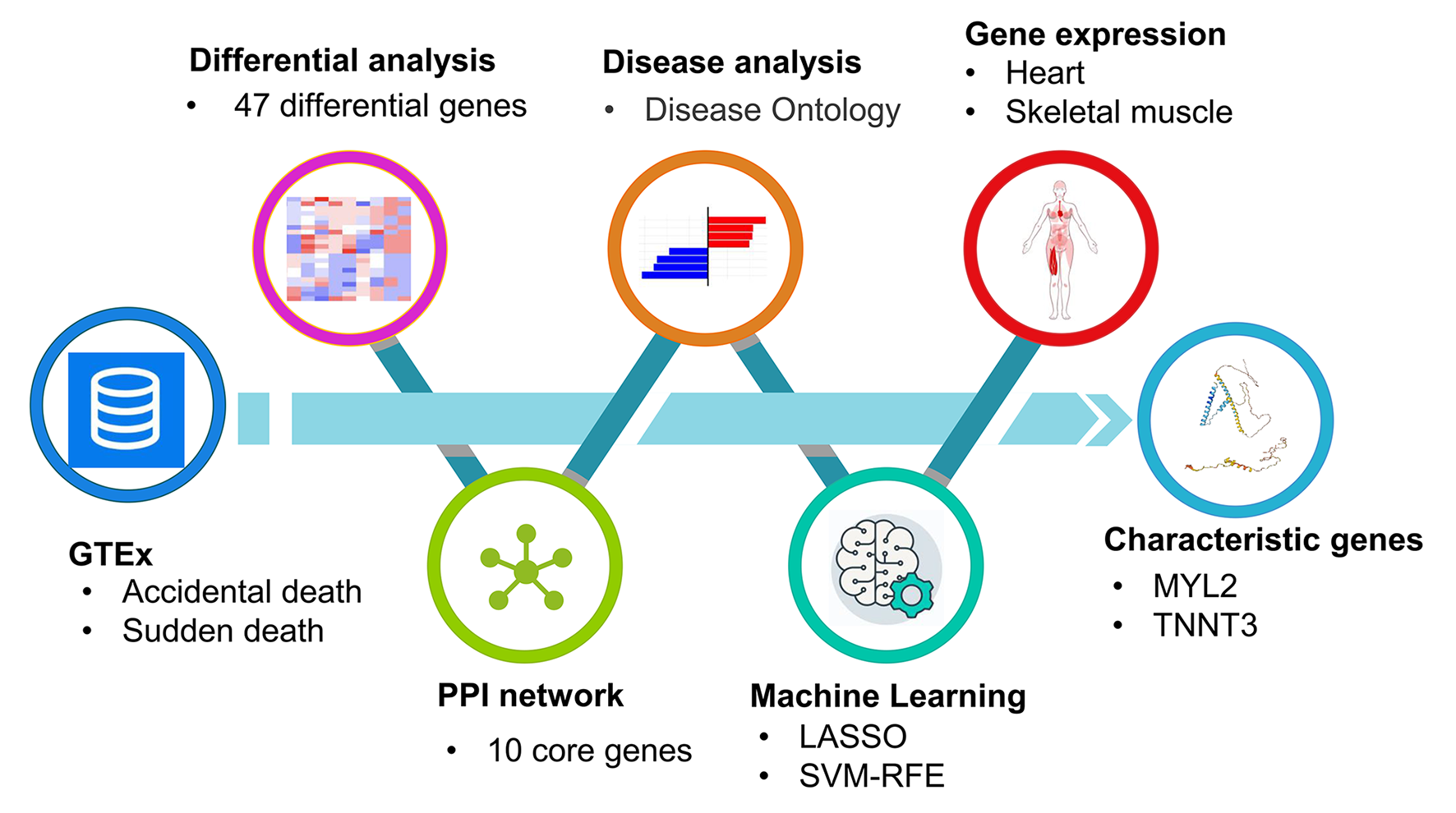

Supplement: Supplementary Figure 1 — Graphical abstract. [file Image_1.TIF]
